# Characterization of Chitosan Nanofiber Sheets for Antifungal Application

**DOI:** 10.3390/ijms161125947

**Published:** 2015-11-02

**Authors:** Mayumi Egusa, Ryo Iwamoto, Hironori Izawa, Minoru Morimoto, Hiroyuki Saimoto, Hironori Kaminaka, Shinsuke Ifuku

**Affiliations:** 1 Department of Chemistry and Biotechnology, Graduate School of Engineering, Tottori University, 4-101 Koyama-cho Minami, Tottori 680-8552, Japan; gonta927@hotmail.com (M.E.); M15T4003M@edu.tottori-u.ac.jp (R.I.); h-izawa@chem.tottori-u.ac.jp (H.I.); morimoto@chem.tottori-u.ac.jp (M.M.); saimoto@chem.tottori-u.ac.jp (H.S.); 2 Faculty of Agriculture, Tottori University, 4-101 Koyama-cho Minami, Tottori 680-8553, Japan; kaminaka@muses.tottori-u.ac.jp

**Keywords:** chitosan nanofibers, surface-deacetylated chitin nanofibers, antifungal activity, dermatophyte

## Abstract

Chitosan produced by the deacetylation of chitin is a cationic polymer with antimicrobial properties. In this study, we demonstrate the improvement of chitosan properties by nanofibrillation. Nanofiber sheets were prepared from nanofibrillated chitosan under neutral conditions. The Young’s modulus and tensile strength of the chitosan NF sheets were higher than those of the chitosan sheets prepared from dissolving chitosan in acetic acid. The chitosan NF sheets showed strong mycelial growth inhibition against dermatophytes *Microsporum* and *Trichophyton*. Moreover, the chitosan NF sheets exhibited resistance to degradation by the fungi, suggesting potentials long-lasting usage. In addition, surface-deacetylated chitin nanofiber (SDCNF) sheets were prepared. The SDCNF sheet had a high Young’s modulus and tensile strength and showed antifungal activity to dermatophytes. These data indicate that nanofibrillation improved the properties of chitosan. Thus, chitosan NF and SDCNF sheets are useful candidates for antimicrobial materials.

## 1. Introduction

Chitosan, produced by the deacetylation of chitin, is a cationic linear polysaccharide [1,2]. Due to its biocompatibility and biodegradability, chitosan has great potential in various fields, and thus chemical and structural modifications have been made to widen its applicability [2,3,4,5,6,7]. We recently developed chitosan nanofiber (NF) and chitin NF (CNF) using the Star Burst system, which employs high-pressure water-jet technology [8,9,10]. NF has a highly uniform structure of 10–20 nm diameters and shows high dispersibility in water due to its submicron size and high surface-to-volume ratio [11]. Furthermore, we have reported surface-deacetylated chitin nanofibers (SDCNF) [12]. Surface-deacetylated chitin was prepared from chitin, whose surface had been transformed into chitosan by deacetylation, and the core part was maintained as chitin crystal. These techniques may help to widen the range of usability of chitosan: biosensor, filtration applications to water and air purification, drug delivery, wound dressing, tissue engineering [3,4,5,6,7].

Moreover, chitosan shows antimicrobial activity against a wide range of microorganisms, such as algae, bacteria, yeasts, and fungi, so it is of special interest for food, hygiene, and medical applications [6,13]. Chitosan showed antifungal activity against *Alternaria brassicicola*, *A. solani*, *Aspergillus flavus*, *A. niger*, *A. parasiticus*, *Botrytis cinerea*, *Byssochlamys* spp., *Fusarium concentricum*, *F. oxysporum*, *Mucor piriformis*, *M. racemosus*, *Pythium debaryanum*, *Rhizoctonia solani*, *Rhizopus stolonifer*, *Saprolegnia parasitica*, and *Ustilago maydis* [14,15,16,17,18,19,20,21,22,23,24,25,26]. These antifungal activity of chitosan in food and crop protection is well documented [27,28]. On the other hand, major effects of chitosan against gram-positive and negative bacteria are well reported in the area of cosmetic, hygiene and medical care, antifungal activity against *Candida* spp. and dermatophytes have been reported [29,30,31].

Dermatophytosis caused by *Epidermophyton*, *Microsporum*, or *Trichophyton* is the most frequent fungal infection in humans and is an important public health problem [32]. *Microsporum* and *Trichophyton* are human and animal pathogens that infect keratinized body surfaces, such as skin, hair, and nails [33,34,35]. Numerous fungicidal and fungistatic agents are used for the treatment of dermatophytosis. To avoid host damage, the targets of these drugs are fungal specific, such as the fungal cell wall and the enzyme involving its synthesis [35]. However, the emergence of antifungal resistance has been reported and attributed to the modification of the target enzyme by mutation and upregulation of the drug efflux [35]. Because dermatophytosis requires long-term treatment, the ideal antifungal drug should be nontoxic to the host, inexpensive, and eco-friendly. Moreover, the prevention of infection is important for disease control. Several reports suggested the availability of chitosan for biomedical application, and hence chitosan is one of the candidate antimicrobial agents used in antimicrobial textiles to prevent the transmission and expansion of infectious pathogen [4,5,6,36,37,38].

In this study, we demonstrate the antifungal activity to dermatophytes of chitosan NF and SDCNF, the nanofibrillation of which enhanced chitosan properties such as the water dispersibility and mechanical strength of a sheet. Chitosan is nontoxic to mammalian cells, hence chitosan NF and SDCNF should be useful materials in pharmaceuticals, cosmetics, household products, and textiles.

## 2. Results and Discussion

### 2.1. Characterization of Nanofibers (NFs)

**Figure 1:**
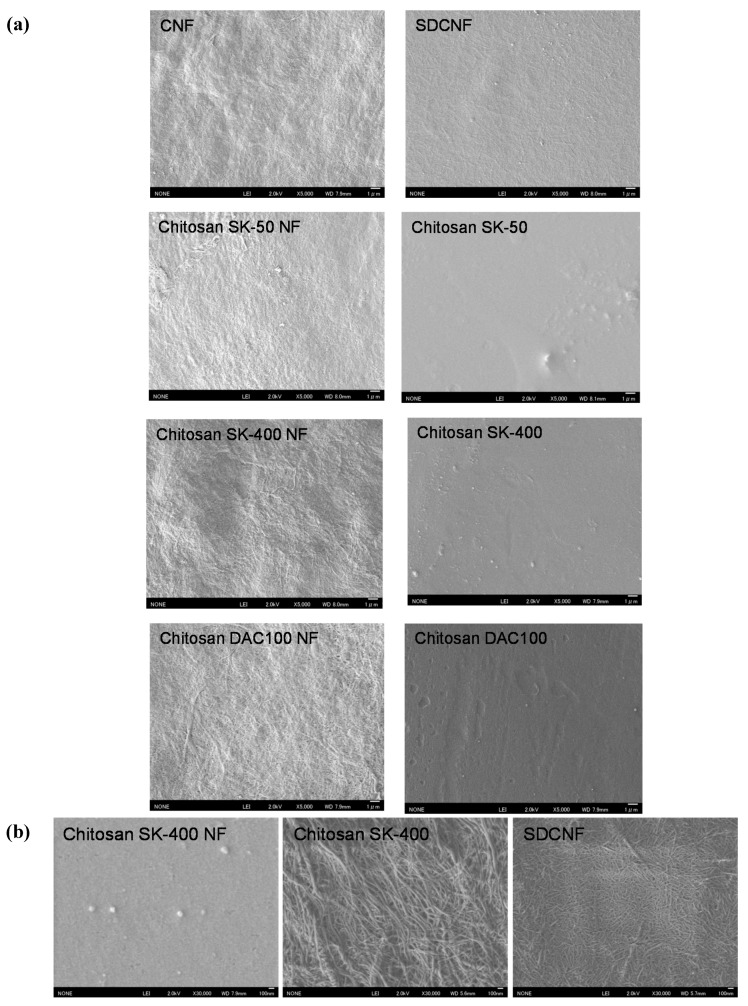
Field emission scanning electron microscopic (FE-SEM) micrographs of sheets. (**a**) SEM micrographs of chitin nanofiber (CNF), surface-deacetylated chitin nanofiber (SDCNF), chitosan NFs and chitosan sheets with low magnification (×5000); (**b**) Representative surface image of chitosan SK-400 NF, Chitosan SK400, and SDCNF sheets with high magnification (×30,000).

**Figure 2:**
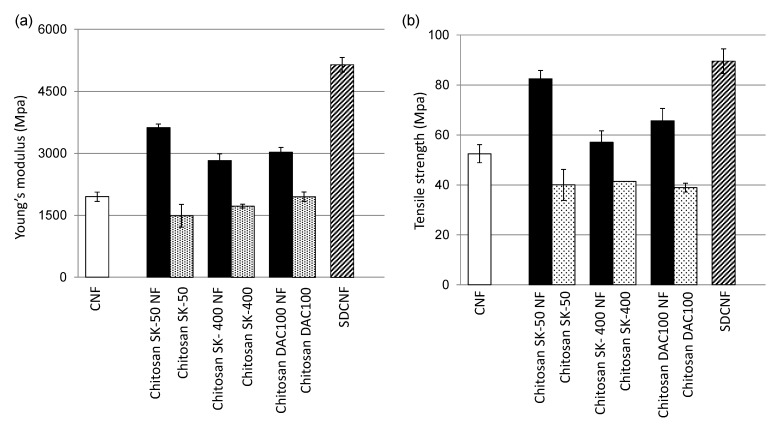
Mechanical properties of nanofiber (NF) sheets. (**a**) Tensile Young’s modulus of NF sheets and (**b**) fracture strength of NF sheets. CNF, chitin nanofiber; SDCNF, surface-deacetylated CNF.

We prepared chitosan NF using the Star Burst system, which employs high-pressure water-jet technology [8,9,10]. Chitin or chitosan dispersed in water were passed through waterjet system equipped with a ball-collision chamber and then slurry was ejected from a small nozzle under high pressure. After mechanical treatments, the NFs became thinner as the number of treatments increased. Although chitosan is insoluble in water, chitosan NF shows high dispersibility in water due to its submicron size and high surface-to-volume ratio. Table 1 shows the properties of the chitosan and chitosan NF materials prepared in this work. It has been reported that antifungal activity depends on degrees of deacetylation (DDA) and molecular weight (*M*w), and thus chitosan NF sheets were prepared from chitosan with different DDA and *M*w [15,39,40]. *M*w of commercial chitosan powder SK-50, SK-400, and DAC100 was 533 kDa, 4612 kDa, and 235 kDa respectively (Table 1). DDA of SK-50 and SK-400 were more than 80%, and DDA of DAC100 was 100%. Mechanical treatment did not affect DDA, but *M*w was decreased (Table 1). It was reported that a large number of passes causes depolymerization [9,10]. Interestingly, the surfaces of the chitosan NFs and SDCNF were positively charged, even though these were prepared under neutral conditions. Figure 1 shows surface image of sheets. The chitosan SK-50, SK-400, and DAC100 sheets prepared from chitosan dissolved in aqueous acetic acid had a smooth surface. On the other hand, the surface of the chitosan NF, SDCNF, and CNF sheets which consists of lamination layers of NFs, showed a fibrous morphology (Figure 1A,B). Figure 2 show the tensile Young’s moduli and fracture strengths of the NF sheets. Both Young’s moduli and the fracture strengths of the chitosan NFs were higher than those of chitosan sheets. Moreover, SDCNF, whose surface was chitosan and whose core part was maintained as chitin crystal, showed the best mechanical properties. Chitosan NF and SDCNF showed low thermal expansion and high density [9,10,12]. These results indicate that the mechanical properties of chitosan were improved by nanofibrillation.

**Table 1:** Properties of chitosan materials.

Sheets	DDA (%)	*M*w (kDa)	Surface Charge
CNF	<5	51	±0
Chitosan SK-50 NF	>80	200	+
Chitosan SK-400 NF	>80	368	+
Chitosan DAC 100 NF	100	10	+
Chitosan SK-50	>80	533	NT
Chitosan SK-400	>80	4612	NT
Chitosan DAC 100	100	235	NT
SDCNF	18	NT	+

DDA, degree of deacetylation; NT, not tested. DDA and *M*w were provided by manufacturer and surface charge was defined by zeta potential measurement.

### 2.2. Antifungal Effects of Chitosan NFs and Surface-Deacetylated Chitin NF (SDCNF)

To assess antifungal effects, the mycelial growth of dermatophytes on mineral salt agar plates was measured (Table 2). CNF had no antifungal activity, whereas chitosan, chitosan NF, and SDCNF sheets inhibited fungal growth (Table 2). No antifungal effect was observed on any sheets to *Trichophyton rubrum*. The mycelial growth of *Microsporum gypseum* was highly inhibited on chitosan NF sheets, and the growth of *M. canis*, *T. mentagrophytes*, and *T. tonsurans* on chitosan sheets was comparable to that on chitosan NF sheets. It was reported that DDA and *M*w affect antifungal activity against *A. solani*, *F. concentricum*, *F. oxysporum*, and *R. solani* [15,20,23,24,26]. According to these studies, there were no general trends in antifungal activity relating to high or low DDA or *M*w, and efficacy of chitosan depended on the particular type of fungus. In our study, there were no significant differences among the sheets (Table 2). Moreover, the comparison of *M*w of NF sheets was inappropriate due to its depolymerization during mechanical nanofibrillation processes. It was suggested that the antimicrobial activity of chitosan in solid state was influenced by surface morphology, size, hydrophilicity, electric charge, and DDA rather than *M*w [13]. Because the surface of a chitosan NF sheet has a fibrous morphology with a high surface-to-volume ratio (Figure 1), it might increase contact between fungi and protonated amino group in chitosan NFs, thereby affecting antifungal activity. Particle size and shape reportedly affect the antibacterial activity of membranes prepared from chitosan particles [40]. Although the exact mechanism underlying the antimicrobial action of chitosan is not fully understood, the most accepted model is that interaction between positively charged chitosan molecules and negatively charged microbial cell membranes, leading to the leakage of intracellular constituents [13,41,42]. Several reports showed that chitosan induced morphological change in the fungal hyphae and spores including swellings and convolutions, and cellular disorganization [17,18,22,23,24,25,26]. On the other hand, another mode of action has indicated that chitosan acts as a chelating agent [16,19]. Therefore, it is conceivable that essential minerals and nutrients binding to chitosan NF and SDCNF might be unavailable for fungal growth. Further investigation will be needed to determine the mode of action of chitosan to dermatophytes.

**Table 2:** Antifungal activity of chitosan nanofibers (NFs) on mycelial growth of dermatophytes.

Sheets	Colony Diameter (mm)				
	*M. canis*	*M. gypseum*	*T. mentagrophytes*	*T. rubrum*	*T. tonsurans*
Blank	2.4 ± 0.05 ^a^	2.4 ± 0.05 ^a^	1.3 ± 0.01 ^a^	1.8 ± 0.02 ^a^	1.7 ± 0.04 ^a^
CNF	1.6 ± 0.06 ^b^	3.0 ± 0.07 ^b^	1.5 ± 0.03 ^b^	1.6 ± 0.04 ^a^	1.7 ± 0.06 ^a^
Chitosan SK-50 NF	1.4 ± 0.02 ^b^	1.0 ± 0.0 ^c^	0.5 ± 0.05 ^cd^	1.9 ± 0.03 ^a^	0.4 ± 0.01 ^bc^
Chitosan SK-400 NF	1.1 ± 0.01 ^bc^	0.9 ± 0.04 ^c^	0.5 ± 0.02 ^c^	1.5 ± 0.2 ^a^	0.5 ± 0.01 ^bc^
Chitosan DAC100 NF	1.2 ± 0.08 ^bc^	0.8 ± 0.02 ^c^	0.5 ± 0.01 ^cd^	1.5 ± 0.04 ^a^	0.4 ± 0.02 ^bc^
Chitosan SK-50	1.7 ± 0.2 ^b^	2.6 ± 0.2 ^ab^	0.4 ± 0.01 ^d^	2.0 ± 0.09 ^a^	0.4 ± 0.02 ^bc^
Chitosan SK-400	1.1 ± 0.3 ^bc^	2.2 ± 0.2 ^a^	0.5 ± 0.01 ^cd^	1.8 ± 0.1 ^a^	0.4 ± 0.02 ^b^
Chitosan DAC100	0.8 ± 0.08 ^c^	2.5 ± 0.2 ^ab^	1.3 ± 0.01 ^cd^	1.5 ± 0.1 ^a^	0.5 ± 0.03 ^c^
SDCNF	1.6 ± 0.1 ^b^	1.0 ± 0.06 ^ac^	0.7 ± 0.02 ^e^	1.8 ± 0.1 ^a^	0.7 ± 0.03 ^d^

Data represent the mean of three independent experiments and standard error. Means with the same letter are not significantly different according to Tukey’s test (*p* < 0.05). CNF, chitin NF; SDCNF, surface-deacetylated CNF.

### 2.3. Resistance of Chitosan NFs and SDCNF against Fungal Degradation

**Figure 3:**
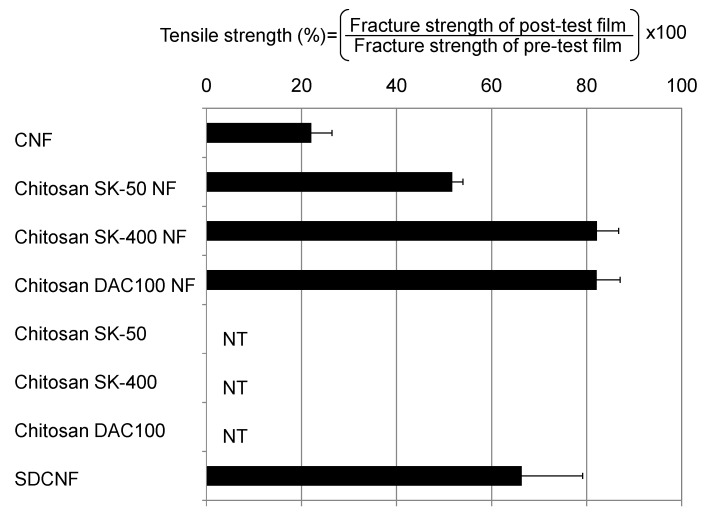
Tensile strength of sheets after the antifungal test. The sheets were harvested from agar plates after the antifungal test and their fracture strength was measured. Data represent the mean of three independent experiments with all five pathogens, and error bars indicate standard error. NT, not tested; CNF, chitin NF; SDCNF, surface-deacetylated CNF.

Since the mineral salt agar plate is a starvation medium, mycelial growth indicates the fungal assimilation of sheets. Dermatophytes secrete a great variety of enzymes in order to obtain nutrients such as carbon, nitrogen, phosphorus, and sulfur from host tissue [33]. Pathogenic fungi generally secrete more enzymes than nonpathogenic fungi, and this is associated with its virulence [33,35]. To assess the resistance of chitosan NF sheets against fungal degradation, the Young’s moduli and the tensile strengths of the sheets were compared from before to after the antifungal tests. Since the Chitosan sheets were fragile, none of chitosan sheets were recovered from the agar plates after the antifungal test (Figure 3). On the other hand, NF sheets were able to be harvested, and chitosan NF sheets and SDCNF showed high tensile strength than control CNF sheets (Figure 3). CNF had no antifungal effect and allowed marked fungal proliferation, which might be accompanied by active secretion of enzymes. Thus, CNF showed low tensile strength after the antifungal test. The chitosan, chitosan NF, and SDCNF sheets showed antifungal activity, indicating fungal biological activity may have been depressed. Yet, the chitosan sheets were degraded. These results indicated that the morphology of a sheet surface seems to affect the resistance against fungal degradation. The attachment of degrading enzymes to the chitosan molecule might be facile on the chitosan sheets than on the chitosan NF sheets due to the fiber-bundle structure of the NF. These results indicated that the chitosan NF and SDCNF sheets exhibited resistance to degradation by the fungi, suggesting their potential for long-lasting use.

## 3. Experimental Section

### 3.1. Preparation of NF

Chitin powder (TC-L) from crab shells and chitosan powder (SK-50, SK-400, and DAC100) were purchased from Koyo Chemical (Tottori, Japan). CNF was prepared without acetic acid as described previously [8]. In brief, dry chitin powder was dispersed in water at 1 wt % and passed through a high-pressure water-jet system (Star Burst Mini, HJP-25001S, Sugino Machine, Toyama, Japan) equipped with a ball-collision chamber for mechanical disintegration. SDCNF was prepared as described previously [12] and modified as follows. Chitin powder was stirred in 20% (*w*/*w*) NaOH for 6 h at 150 °C under a nitrogen atmosphere. After deacetylation, the supernatant was removed and the precipitate was washed with distilled water. Surface-deacetylated chitin was dispersed in aqueous acetic acid (0.5% (*w*/*w*)) to remove any soluble products and then thoroughly washed with distilled water by centrifugation. The sample was passed through a grinder (MKCA6–3; Masuko Sangyo Co., Kawaguchi, Japan) once at 1500 rpm and then passed through a high-pressure water-jet system as described above. Chitosan NFs were prepared as described previously [9,10].

### 3.2. Preparation of Nanofiber Sheets

The above-prepared NFs dispersed in water were vacuum-filtered using a hydrophilic polytetrafluoroethylene membrane filter (Millipore, pore size: 0.2 μm) and washed with distilled water and ethanol. The obtained NF sheets were hot-pressed at 100 °C for 20 min to obtain a dried sheet consisting of 200 mg of chitin or chitosan.

### 3.3. Preparation of Chitosan Sheets

Chitosan was dissolved in aqueous acetic acid (1% (*w*/*w*)). The chitosan solution was poured into glass petri dishes coated with a release agent and dried under ambient conditions (40 °C, 3 days). The obtained chitosan sheets were neutralized by immersion in 0.5% (*w*/*w*) NaOH for 5 min and then were washed in distilled water. The sheets were hot-pressed at 100 °C for 20 min to obtain a dried sheet consisting of 200 mg of chitosan.

### 3.4. Characterization of Nanofibers

For field emission scanning electron microscopic (FE-SEM) observation, the sample was coated with an approximately 2 nm layer of Pt by an ion sputter coater and observed by a JSM-6700F scanning electron microscope (JEOL Ltd., Tokyo, Japan) operating at 2.0 kV. The Young’s modulus and tensile strength of each NF or chitosan sheet (3 cm in length and 1 cm in width) were measured using a universal testing instrument (AG-X, Shimadzu, Tokyo, Japan). The zeta potentials of the NF dispersants were measured with a Zeta-Potential & Particle Size Analyzer (ELSZ-1000ZS, Otsuka Electronics, Osaka, Japan).

### 3.5. Fungal Material

Dermatophytes including *M. canis* (NBRC9182), *M. gypseum* (NBRC5948), *T. mentagrophytes* (NBRC5466), *T. rubrum* (NBRC5807), and *T. tonsurans* (NBRC5946) were obtained from NBRC (NITE Biological Resource Center, Chiba, Japan). All fungi were maintained on Sabouraud agar medium.

### 3.6. Antifungal Activity Test

NF sheets or chitosan sheets (cut into 3 cm × 3 cm pieces) were placed on mineral salt agar plates (3 g NH_4_NO_3_, 1 g KH_2_PO_4_, 0.5 g MgSO_4_·7H_2_O, 0.25g KCl, 0.002 g FeSO_4_·7H_2_O, 20 g Agar per liter), and then a piece of fungal block (diameter 2 mm) was placed on each sheet. The plates were placed under 25 °C, and the colony diameter was measured at the appropriate time.

## 4. Conclusions

Chitosan NF and SDCNF sheets were prepared using a high-pressure waterjet system under neutral conditions. The NF sheets exhibited improved mechanical properties and inhibited mycelial growth against dermatophytes. Moreover, the chitosan NF and SDCNF sheets exhibited resistance to degradation by the fungi, suggesting their potential for long-lasting use. It is apparent that public concern for environmental and biological systems is growing, and thus ideal antimicrobial products should be safe for human and animals and eco-friendly. Because chitosan shows favorable biological properties such as non-toxicity, biocompatibility, biodegradability, and antimicrobial activity, chitosan is promising candidates as active natural material. Moreover there are many evidence of the beneficial effect of chitin and chitosan for biomedical area and these nano-fibrillation materials enhance their efficacy. Chitosan NF and SDCNF nanofibers are promising candidates for in pharmaceuticals, cosmetics, household products, and textiles.
